# Solubility, Stability, and Avidity of Recombinant Antibody Fragments Expressed in Microorganisms

**DOI:** 10.3389/fmicb.2020.01927

**Published:** 2020-09-25

**Authors:** Tae Hyun Kang, Baik Lin Seong

**Affiliations:** ^1^Biopharmaceutical Chemistry Major, School of Applied Chemistry, Kookmin University, Seoul, South Korea; ^2^Department of Biotechnology, College of Life Science and Biotechnology, Yonsei University, Seoul, South Korea; ^3^Vaccine Innovative Technology ALliance (VITAL)-Korea, Yonsei University, Seoul, South Korea

**Keywords:** antibody fragments, solubility, stability, bacterial expression, scFv

## Abstract

Solubility of recombinant proteins (i.e., the extent of soluble versus insoluble expression in heterogeneous hosts) is the first checkpoint criterion for determining recombinant protein quality. However, even soluble proteins often fail to represent functional activity because of the involvement of non-functional, misfolded, soluble aggregates, which compromise recombinant protein quality. Therefore, screening of solubility and folding competence is crucial for improving the quality of recombinant proteins, especially for therapeutic applications. The issue is often highlighted especially in bacterial recombinant hosts, since bacterial cytoplasm does not provide an optimal environment for the folding of target proteins of mammalian origin. Antibody fragments, such as single-chain variable fragment (scFv), single-chain antibody (scAb), and fragment antigen binding (Fab), have been utilized for numerous applications such as diagnostics, research reagents, or therapeutics. Antibody fragments can be efficiently expressed in microorganisms so that they offer several advantages for diagnostic applications such as low cost and high yield. However, scFv and scAb fragments have generally lower stability to thermal stress than full-length antibodies, necessitating a judicious combination of designer antibodies, and bacterial hosts harnessed with robust chaperone function. In this review, we discuss efforts on not only the production of antibodies or antibody fragments in microorganisms but also scFv stabilization via (i) directed evolution of variants with increased stability using display systems, (ii) stabilization of the interface between variable regions of heavy (V_*H*_) and light (V_*L*_) chains through the introduction of a non-native covalent bond between the two chains, (iii) rational engineering of V_*H*_-V_*L*_ pair, based on the structure, and (iv) computational approaches. We also review recent advances in stability design, increase in avidity by multimerization, and maintaining the functional competence of chimeric proteins prompted by various types of chaperones.

## Introduction

**FIGURE 1:**
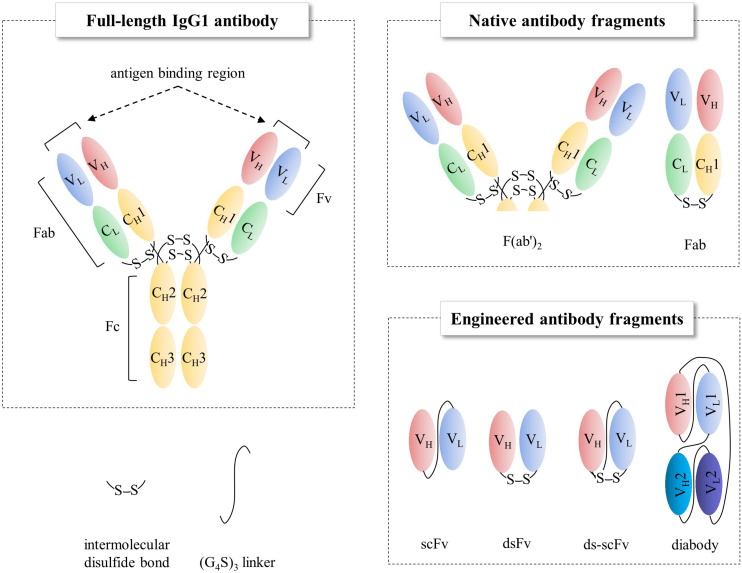
Schematic representations of full-length IgG1 antibody and antibody fragments. Fab, fragment antigen binding; Fv, fragment variable; Fc, fragment crystallizable; scFv, single-chain Fv; dsFv, disulfide-stabilized Fv.

Antibodies are widely used for medical applications such as disease diagnosis and therapy (Grilo and Mantalaris, 2019). Valuable pharmaceutical properties of antibodies such as high affinity to their target molecules have led to them becoming constituted as key materials not only in antibody-based biosensors, which offer the promise of in-depth target detection capacity (Holliger and Hudson, 2005; Saerens et al., 2008; Conroy et al., 2009) but also in antibody-based proteomics, which provides insights into cancer disease states via cancer biomarker discovery (Brennan et al., 2010). In addition to the intrinsic property, industrial applications require high productivity and long shelf-life from thermal stress, organic solvents, and other stresses than physiological conditions. However, production of full-length antibodies cost extremely high, as they are typically expressed in mammalian cell lines such as Chinese hamster ovary (CHO) or NS0 murine myeloma cell lines (de la Cruz Edmonds et al., 2006; Huang et al., 2007; Liu et al., 2008; Grilo and Mantalaris, 2019) due to N-glycan heterogeneity among different species (Jefferis, 2009) and also the complex disulfide bond pattern; hence, the biopharmaceutics industry has devoted immense resources on its production processes (Chartrain and Chu, 2008). Instead, the single-chain variable fragment (scFv, Figure 1), a rational polypeptide design, consisting only of variable regions from heavy (V_*H*_) and light (V_*L*_) chains, joined together by a linker, not only maintains antigen binding capacity (Humphreys, 2003; Andersen and Reilly, 2004) but also can easily be produced in prokaryotes, such as *Escherichia coli* (*E. coli*) or *Brevibacillus choshinensis* (*B. chosinensis*) (Hu et al., 2017), along with high yield, which keeps the cost of production low (Power and Hudson, 2000; Terpe, 2006; Rosano and Ceccarelli, 2014; Gupta and Shukla, 2017). Despite the advantages of scFvs, they have a few drawbacks that limit their therapeutic potential, such as (i) deteriorated stability because of their propensity to readily aggregate under thermal stress (Jager and Pluckthun, 1999a; Demarest and Glaser, 2008); (ii) a short serum half-life of <1 day compared to 3 weeks for full-length immunoglobulin G (IgG)1, IgG2, and IgG4 antibodies (Kang and Jung, 2020); and (iii) reduced affinity compared to the full-length antibody counterpart. Therefore, scFv format is suitable for limited cases, such as macular degeneration or blood-related diseases (Table 1).

**TABLE 1:** FDA approved therapeutic antibody fragments.

Name^1^	Brand name^2^	Company^3^	Target	Format	Indication^4^	Year approved^5^	Host^6^
Abciximab	Reopro	Janssen Biotech, Inc.	GPIIb/IIIa	Chimeric IgG1 Fab	Prevention of blood clots in angioplasty	1994	Mammalian
Ranibizumab	Lucentis	Genentech, Inc.	VEGF	Humanized IgG1 Fab	Macular degeneration	2006	*E. coli*
Certolizumab pegol	Cimzia	UCB, Inc.	TNF	Humanized Fab, pegylated	Crohn disease	2008	*E. coli*
Blinatumomab	Blincyto	Amgen Inc.	CD19, CD3	Murine bispecific tandem scFv	Acute lymphoblastic leukemia	2014	*E. coli*
Idarucizumab	Praxbind	Boehringer Ingelheim Pharmaceuticals, Inc.	Dabigatran	Humanized Fab	Reversal of dabigatran-induced anticoagulation	2015	CHO^7^
Moxetumomab pasudotox	Lumoxiti	AstraZeneca Pharmaceuticals LP	CD22	Murine IgG1 dsFv immunotoxin	Hairy cell leukemia	2018	*E. coli*
Caplacizumab	Cablivi	Ablynx N.V.	von Willebrand factor	Humanized Nanobody	Acquired thrombotic thrombocytopenic purpura	2019	*E. coli*
Brolucizumab	Beovu	Novartis Pharmaceuticals Corporation	VEGF-A	Humanized scFv	Neovascular age-related macular degeneration	2019	*E. coli*

*^1^Name: International non- proprietary names. ^2^Brand name: commercial names. ^3^Company: companies developed the antibody fragment drugs. ^4^Indication: indication first approved by the FDA. ^5^Year approved: the first year approved by the FDA. ^6^Host: expression cell-lines. ^7^CHO: Chinese Hamster Ovary cell-line. This table was classified using data from “The Antibody Society (2020)” (Antibody Society, 2020).*

**FIGURE 2:**
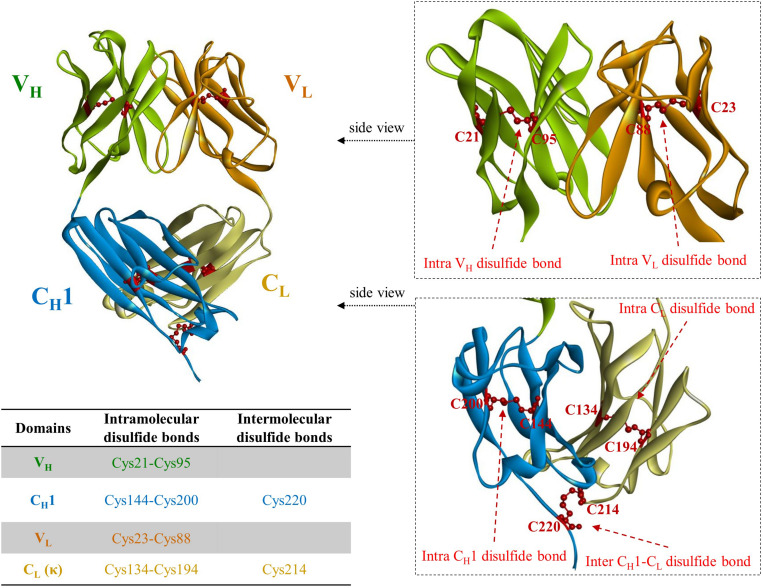
A crystal structure of fragment antigen binding (Fab) region of canakinumab, a human monoclonal antibody neutralizing IL-1β (PDB ID: 5BVJ). V_*H*_ regions are colored in light green; C_*H*_1 in light blue; V_*L*_ in orange; C_*L*_ in cyan. Cysteine residues involved in disulfide bonds are partially depicted in red ball and stick model. The only intermolecular disulfide bonds between heavy and light chain of IgG molecule is present on the cysteine residues in C_*H*_1 (Cys220) and C_*L*_ (Cys214), respectively. The positions of the cysteine residues vary among different V_*H*_ and V_*L*_ due to the differences in CDR length.

Immunoglobulin G, the most abundant monoclonal antibody (mAb) isotype in serum is composed of two antigen binding fragments (Fab) and one homodimeric fragment crystallizable (Fc) domain that contribute to the overall stability of the molecule (Figure 1; Kang and Jung, 2019; Saunders, 2019). Since Fab of an IgG becomes more sensitive to the heat denaturation when Fc region is removed (Tischenko et al., 1982; Vermeer and Norde, 2000; Ionescu et al., 2008), researchers have tried to engineer Fab to stabilize the interactions between constant heavy 1 (C_*H*_1) and constant light (C_*L*_) chains in order to obviate the need for using mammalian host cells for the expression of full-length antibodies because of *N*-glycan on the Fc region. This requires immense resources such as expensive media, facilities to maintain germfree conditions, and time. However, limited successes have been made (Demarest et al., 2006; Teerinen et al., 2006). Further elimination of C_*H*_1-C_*L*_ pair in Fab, resulting in fragment variable (Fv), significantly discounts thermodynamic stability (Webber et al., 1995; Jager and Pluckthun, 1999b). This is presumably due to the unnatural exposure of the lower V_*L*_ and V_*H*_ regions, flanking C_*H*_1 and C_*L*_, where hydrophobic interaction used to contribute to the stability as a whole as well as the absence of the contribution of C_*H*_1, which controls the assembly of heavy and light chains of the whole IgG molecule (Feige et al., 2009). The only light-heavy intermolecular disulfide bond in native IgG antibodies on the residues Cys220 in C_*H*_1 and Cys214 in C_*L*_ of Fab region (Figure 2 in canakinumab; PDB ID of 5BVJ) contributes to the thermodynamic stability of the whole Fab fragment. In addition, intramolecular disulfide bonds in both the V_*H*_ and V_*L*_ regions (Figure 2) is critical in the thermodynamic stability because elimination of them significantly enhanced the propensity of scFv aggregation (Montoliu-Gaya et al., 2017). In this article we review efforts on increasing expression yield as well as protein stability of antibody fragment and recent diverse designs of antibody fragments.

## Production of Antibody or Antibody Fragments in Bacteria

To reduce the cost of production of antibodies, researchers in both academia and industry put enormous efforts on elevating expression yield of IgG antibody or its fragment by (i) engineering expression plasmids, i.e., rhamnose-inducible expression system (Petrus et al., 2019) or comprehensive optimization via high-throughput screening (Makino et al., 2011), (ii) engineering global sigma factor RhoD, which regulates more than 1,000 gene expressions (McKenna et al., 2019), and (iii) devising bacterial strains capable of forming disulfide bonds in cytoplasm such as CyDisCo (Gaciarz and Ruddock, 2017) or SHuffle (Lobstein et al., 2012; Robinson et al., 2015; Yusakul et al., 2017). Despite the advantages, drawbacks limiting its potential are (i) the low stability of scFvs, known for their propensity to readily aggregate under thermal stress (Jager and Pluckthun, 1999a; Demarest and Glaser, 2008), (ii) absence of glycosylation machinery, (iii) lack of efficient secretory mechanism as compared to yeast or animal cells, functionally limited protein trafficking machinery from the cytoplasm to the periplasmic space or to the outside of the cells, and (iv) overproduction of acetic acid byproduct during fermentation (Holms, 1986; Wong et al., 2008).

## Engineering Intrinsic Stability of scFvs: Directed Evolution, Rational Design, and Computational Approaches

Antibody fragments can be expressed in several compartments in *E. coli*: mostly as inclusion bodies in the cytoplasm, or as soluble forms displayed on (i) the inner membrane, (ii) in the periplasmic space, (iii) on the outer membrane, and/or (iv) outside the bacterium, facilitated by various signal sequences, such as outer membrane protein A (OmpA), pectate lyase B (PelB), or new lipoprotein A (NlpA) (Tseng et al., 2009; Frenzel et al., 2013; Khodabakhsh et al., 2013; Levy et al., 2013; DePalma, 2014; Mizukami et al., 2018). To overcome the drawbacks of scFvs, which comprise only V_*H*_ and V_*L*_ antigen-binding domains, to reduce the protein size in order to increase protein production but maintain high target molecule affinity, researchers have engineered scFvs with resistance to aggregation and enhanced intrinsic stability of antibody fragments. Table 2 summarizes the engineering efforts.

**TABLE 2:** Summary of stability and avidity engineering of scFvs in bacteria.

Classification	Group	Institute	Methodology	References
Directed evolution	Gregory Winter	University of Cambridge	Phage display	Jespers et al., 2004
	Daniel Christ	Garvan Institute of Medical Research	Phage display	Dudgeon et al., 2012
	Dane Wittrup	MIT	Yeast display	Graff et al., 2004; Chao et al., 2006
	Scott Glaser	Biogen Idec	Bacterial screening	Miller et al., 2010
Rational design	Ira Pastan	NIH	Non-native disulfide bond	Brinkmann et al., 1993
	Andreas Plückthun	University of Zurich	Disrupting the hydrophobic patches	Nieba et al., 1997
	Wei Chen	Jiangnan University	Non-native disulfide bond	Zhao et al., 2010
	Ning Deng	Jinan University	Non-native disulfide bond	Cai et al., 2016
	Hiroshi Morioka	Kumamoto University	Cyclization of scFv	Yamauchi et al., 2019
	Alexey Lugovskoy	Merrimack Pharmaceuticals	Tyrosine substitution	Zhang et al., 2015
	An-Suei Yang	National Defense Medical Center in Taipei	Hydrophobic domains	Hsu et al., 2014
	Robin Curtis	University of Manchester	Substitution of Arginine	Austerberry et al., 2019
	Boris Steipe	University of Toronto	Consensus-based	Steipe, 2004
	Alexey Lugovskoy	Biogen Idec	Consensus-based	Jordan et al., 2009
Computation	Andreas Plückthun	University of Zurich	Structure-based	Ewert et al., 2003, 2004
	David R. Liu	Harvard University	Supercharging	Lawrence et al., 2007
	Andrew Ellington	University of Texas at Austin	Supercharging	Miklos et al., 2012
	Peter Bowers	AnaptysBio	Integrative approach	McConnell et al., 2013; McConnell et al., 2014
	George Georgiou and Andrew Ellington	University of Texas at Austin	Rosetta modeling	Lee et al., 2019
Fusion partner	Yechen Xiao	Jilin University	SUMO	Liu et al., 2015
	Junichi Takagi	Osaka University	Fv-clasp	Arimori et al., 2017
Chaperone coexpression	Shihua Wang	Fujian Agriculture and Forestry University	Skp	Wang et al., 2013
Multimerization	Sergey Kipriyanov	Affimed Therapeutics AG	Diabody: bispecific	Le Gall et al., 2004
	C Ronald Geyer	University of Saskatchewan	SpyCatcher: trispecific	Alam et al., 2018

### Directed Evolution

Greg Winter et al., utilized phage display directed evolution methodology to isolate V_*H*_ variants that are more resistant to heat denaturation (Jespers et al., 2004). They further engineered V_*H*_ and identified a key residue, Arg28 in V_*H*_ that renders resistance to heat and acid aggregation (Famm et al., 2008). Daniel Christ’s group at the Garvan Institute of Medical Research selected critical residues for antigen binding in both V_*H*_ and V_*L*_ and constructed a phage library introducing aspartate or glutamate in those residues to screen for heat resistance. The isolated variants resulted in not only enhanced biophysical property but also structural conservation (Dudgeon et al., 2012). Dane Wittrup’s group at MIT devised a yeast surface display system to isolate scFv variants with high affinity to antigen and increased stability by constructing yeast mutant libraries, expressing scFv on the cell surface, followed by successive rounds of flow cytometry sorting (Graff et al., 2004; Chao et al., 2006). Brian Miller et al., at Biogen Idec, Inc. used sequence- and structure-based analyses to devise a high-throughput screening methodology that measure scFv extracellularly expressed by *E. coli*. This screening methodology resulted in enhanced melting temperature (Tm) by 14°C and additional Tm improvement by 12°C through combination of the resulting variants (Miller et al., 2010).

### Rational Design

Unlike the presence of intermolecular C_*H*_1-C_*L*_ disulfide bond, there is not one in the native V_*H*_-V_*L*_ (Figures 1, 2). Instead of placing a linker between V_*H*_ and V_*L*_ (scFv in Figure 1) creating non-native disulfide bond between V_*H*_ and V_*L*_ via substituting amino acid residues in both framework 2 (FR2) in V_*H*_ and FR4 in V_*L*_ (dsFv; disulfide-stabilized Fv in Figure 1) led to indistinguishable specificity to antigen and similar cytotoxic activity when fused with exotoxin but exhibited superior protein stability at 37°C, compared to scFv counterpart (Brinkmann et al., 1993). Similarly, substitution of Val84 in V_*H*_ to aspartic acid led to not only improved periplasmic production by 25-fold but also decreased the rate of thermally induced aggregation reaction (Nieba et al., 1997). In another study, introduction of Cys44 in V_*H*_ and Cys100 in V_*L*_ in anti-aflatoxin B(1) scFv resulted in improved stability and resistance to protein aggregation (Zhao et al., 2010). Introduction of the disulfide bond to anti-FGF2 diabody (ds-diabody, see section “multimerization” for diabody) also improved biological activity (Cai et al., 2016). This is presumably due to its lower propensity to the open state of V_*H*_-V_*L*_ pair, in contrast to the presence of both assembled and disassembled state in case of scFv where V_*H*_ and V_*L*_ domains are simply connected by a G_4_S linker. These results indicate that bridging V_*H*_ and V_*L*_ by establishing intermolecular disulfide bond formation via cysteine residue incorporation can be a decent strategy for Fv stabilization (Trivedi et al., 2009). Another recent approach incorporating closed state of V_*H*_-V_*L*_ pair is a cyclization of scFv using an enzyme sortase A, which ligate the pair, leading to cyclic scFv: this methodology markedly suppressed aggregation tendency without affecting affinity to antigen (Yamauchi et al., 2019).

Alexey Lugovskoy et al., at Merrimack Pharmaceuticals, Inc. showed that both essential and non-essential tyrosine residues for antigen binding in either CDR or FR can improve the biophysical property of scFv (Zhang et al., 2015). An-Suei Yang et al., at the National Defense Medical Center, Taipei, elucidated the nature of intra- and inter-hydrophobic domains of scFv: the former is flexible and indirectly affects antigen binding, as opposed to the latter affecting antigen binding directly (Hsu et al., 2014). Robin Curtis’s group at the University of Manchester investigated the aggregation propensity of arginine-rich scFv under denaturing condition: substitution of arginine residues in scFv with lysine significantly reduced aggregation (Austerberry et al., 2019). This diverse knowledge of protein nature in scFv may assist engineers with consensus-based design of antibody fragment for generating stabilizing mutations to pre-existing scFvs (Steipe, 2004) or bispecific antibody fragments (Jordan et al., 2009).

### Computational Approaches

Andreas Plückthun’s group at University of Zurich designed a stabilized scFv from human V_*H*_ germline sequences by analyzing hydrophobic core, pairing of hydrogen bonds, clusters of charge, and packing of β-sheets, leading to reduction of ΔG = 20.9 kJ/mol as well as improvement of scFv expression yield by 4-fold (Ewert et al., 2003). Furthermore, they could stabilize scFv by CDR grafting to more stable framework, using a structure-based analysis (Ewert et al., 2004).

Computational web servers, such as Prediction of Immunoglobulin Structure (PIGS) (Marcatili et al., 2008) or Web Antibody Modeling (WAM) (Whitelegg and Rees, 2000), made computational modeling of antibody variable regions possible. Importantly, recent advances in structural knowledge and computational protein modeling such as RosettaDesign accelerated antibody design toward improved antigen affinity as well as physicochemical properties (Borgo and Havranek, 2012; Buck et al., 2012). The homology modeling provides with guidance on not only prediction on the conformations of CDR loops but also V_*H*_-V_*L*_ orientations via energy calculations such as antibody-antigen docking, comparing with known crystal structures (Kuroda et al., 2012). For example, a computational homology modeling significantly improved resistance of scFvs to heat inactivation by supercharging the protein through energy calculations (Lawrence et al., 2007; Miklos et al., 2012). In addition, combinatorial engineering approach, including CDR grafting onto stable frameworks, V_*H*_-V_*L*_ interface stabilization, and *in vitro* somatic hypermutation significantly increased thermal stability of full-length antibody by 10°C, compared to the native IgG1 antibody (McConnell et al., 2013, 2014).

Recent advances in computational methodologies for both antibody sequencing and backbones (Goldenzweig and Fleishman, 2018) and for multistage processing of antibody engineering, capitalizing on computational design and experimental validation cycles (Baran et al., 2017), have enabled successful *de novo* antibody engineering (Chevalier et al., 2017), such as anti-influenza antibodies (Strauch et al., 2017; Sevy et al., 2019). Of note, Georgiou and Ellington at the University of Texas at Austin used the Rosetta modeling program (Sircar et al., 2009; Adolf-Bryfogle et al., 2018) to predict amino acid substitutions for anti-HA33 scFv stabilization and confirmed a melting temperature increase of 4.5°C by antigen-binding enzyme-linked immunosorbent assay (ELISA) after thermal stress for 2 h at 70°C (Lee et al., 2019).

## A Fusion Partner

Tagging of anti-FGFR3 scFv with a solubilizing partner small ubiquitin-related modifier (Sumo), followed by removal of the Sumo protein using Sumo protease, enabled over 95% purity with the yield of 4 mg/L bacterial culture. The resulting anti-FGFR3 scFv has exhibited complete biological activity (Liu et al., 2015). Another example is an “Fv-clasp,” where scFv was fused with anti-parallel coiled coil structure, SRAH domain of human Mst1 with scFv. In addition, introduction of disulfide bond to the Fv greatly enhanced thermal stability and tendency of crystallization. This is presumably due to the shielding of hydrophobic residues exposed in Fv, according to the X-ray crystallography (Arimori et al., 2017). Alternatively, approaches based on the chaperone function of RNAs could also be considered (Choi et al., 2008). Fusion with an RNA-interaction domain (RID) greatly enhances the solubility (i.e., the ratio of soluble versus insoluble expression in heterogeneous hosts) and the overall yield of soluble proteins, harnessed with unique properties of RNAs as chaperone (chaperna; chaperone + RNA) (Kim et al., 2018; Yang et al., 2018), although this approach has not yet been documented for recombinant antibody fragments.

## Engineering the Host Cell: Chaperone Coexpression or Genome-Level Screening

The folding of nascent polypeptides is often assisted by molecular chaperones (Hendrick and Hartl, 1995; Bukau and Horwich, 1998; Hartl and Hayer-Hartl, 2002), although their utility in recombinant expression has been documented only in limited cases (Structural Genomics Consortium et al., 2008). The stability problem associated with V_*H*_-V_*L*_ pair in the scFv molecule can be circumvented through assistance with the chaperone of a pairing vehicle. Coexpression of the chaperone Skp enhanced binding activity of anti-TLH scFv by 3–4 fold, relative to the native counterpart, expressed in *E. coli* (Wang et al., 2013).

## Multimerization

Diabodies not only render bivalency but also enhance stability of Fv by linker design (Le Gall et al., 2004). Introducing covalent bonds between V_*H*_1 and V_*L*_2 as well as V_*H*_2 and V_*L*_1 enables bispecific binding capacity of the two distinct scFv in one molecule (diabody in Figure 1). One example in clinic is blinatumomab (BLINCYTO,^®^ 2014), a bispecific scFv for CD19 and CD3 (Table 1), which functions as a T lymphocyte engager to cancerous B lymphocytes for the treatment of acute lymphoblastc leukemia (Holliger et al., 1993; Mack et al., 1995; Suresh et al., 2014; Wu et al., 2015). In addition, constructing anti-HER3 trivalent scFv using SpyCatcher ligase system enhanced affinity by 12-fold as compared to a monomeric anti-HER3 counterpart (Alam et al., 2018). Another general approach of antibody fragments is the utilization of targeting ligands on nanoparticles in nanomedicine (Richards et al., 2017). Several antibody fragment-based nanoparticles are under clinical trials, including Erbitux-EDVS_pac_, which is a bacteria-derived mini-cell nanoparticle targeting EGFR currently under clinical phase II (Richards et al., 2017). Multimerization of scFvs as nanoparticles, using self-assembling scaffolds via chaperna approach (Kim et al., 2018; Yang et al., 2018) holds promise for further enhancing the avidity and thermostability of recombinant antibody fragments.

## Conclusion

Up to March 2020, the US Food and Drug Administration (FDA) approved eight antibody fragments as drugs, six of which are produced from *E. coli* (75%, Table 1). The six therapeutic antibody fragments, produced by bacteria include ranibizumab (LUCENTIS^®^, 2006), certolizumab pegol (CIMZIA^®^, 2008), blinatumomab (BLINCYTO^®^, 2014), moxetumomab pasudotox (Kreitman and Pastan, 2011; LUMOXITI^TM^, 2018), caplacizumab (CABLIVI^®^, 2019), and brolucizumab (BEOVU^®^, 2019), while those produced by mammalian hosts include abciximab (ReoPro^®^, 1994) and idarucizumab (PRAXBIND^®^, 2015), according to the data from “The Antibody Society” (Antibody Society, 2020).

Microorganisms are favorable expression hosts for antibody fragments, such as scFvs or Fab, in therapeutic applications (Table 1), because of the low production cost and lack of a carbohydrate chain. However, despite these advantages, scFvs expressed in bacteria have neither comparable stability relative to native full-length antibodies nor a comparable production yield of ∼1 g/L in bioreactors (Petrus et al., 2019) relative to mammalian hosts, that is, >10 g/L in CHO cells (Kunert and Reinhart, 2016). Therefore, scientists and engineers in both academia and industry put extensive efforts on increasing production yield as well as protein stability of scFv expressed in bacteria. To obtain improved yield various bacterial expression systems have been developed in terms of vector systems or engineered strains with engineered chaperone molecules.

The low intrinsic solubility and stability of native scFv protein with a relatively shorter shelf-life is a bottleneck for industrial application. To overcome the disadvantages there have been enormous research attempts on stability design via site-directed mutagenesis, generation of non-natural covalent bonds between the heavy and light variable chains, rational design, and recently computer-based engineering or chimeric approaches. Engineering of scFv with respect to increasing the stability lowers both the kinetic complexity in folding process and subsequently the propensity to aggregate into non-functional form. Folding into soluble, functional form with the desired level of avidity is often aided by exploiting the chaperone function of naturally existing molecular chaperones or artificial solubilizing tags. Besides thermodynamic aspects on overall stability, due consideration should be given to the kinetic aspects in *de novo* folding pathway for designer antibody fragments toward improved solubility, thermal stability, and productivity.

## Author Contributions

TK and BS designed and wrote the manuscript. Both authors agreed to be accountable for the content of the work.

## Conflict of Interest

The authors declare that the research was conducted in the absence of any commercial or financial relationships that could be construed as a potential conflict of interest.

## References

[B1] Adolf-Bryfogle J. Kalyuzhniy O. Kubitz M. Weitzner B. D. Hu X. Adachi Y. (2018). RosettaAntibodyDesign (RAbD): a general framework for computational antibody design. *PLoS Comput. Biol.* 14:e1006112. 10.1371/journal.pcbi.1006112 29702641 PMC5942852

[B2] Alam M. K. Brabant M. Viswas R. S. Barreto K. Fonge H. Ronald Geyer C. (2018). A novel synthetic trivalent single chain variable fragment (tri-scFv) construction platform based on the SpyTag/SpyCatcher protein ligase system. *BMC Biotechnol.* 18:55. 10.1186/s12896-018-0466-6 30200951 PMC6131909

[B3] Andersen D. C. Reilly D. E. (2004). Production technologies for monoclonal antibodies and their fragments. *Curr. Opin. Biotechnol.* 15 456–462. 10.1016/j.copbio.2004.08.002 15464378

[B4] Antibody Society (2020). Available online at: https://www.antibodysociety.org/resources/approved-antibodies/ (accessed March 10, 2020).

[B5] Arimori T. Kitago Y. Umitsu M. Fujii Y. Asaki R. Tamura-Kawakami K. (2017). Fv-clasp: an artificially designed small antibody fragment with improved production compatibility, stability, and crystallizability. *Structure* 25 1611–1622. 10.1016/j.str.2017.08.011 28919443

[B6] Austerberry J. I. Thistlethwaite A. Fisher K. Golovanov A. P. Pluen A. Esfandiary R. (2019). Arginine to Lysine mutations increase the aggregation stability of a single-chain variable fragment through unfolded-state interactions. *Biochemistry* 58 3413–3421. 10.1021/acs.biochem.9b00367 31314511

[B7] Baran D. Pszolla M. G. Lapidoth G. D. Norn C. Dym O. Unger T. (2017). Principles for computational design of binding antibodies. *Proc. Natl. Acad. Sci. U.S.A.* 114 10900–10905. 10.1073/pnas.1707171114 28973872 PMC5642698

[B8] BEOVU^®^ (2019). *Prescribing Information Approved by the U.S. Food and Drug Administration.* (U.S. License Number: 1244). East Hanover, NJ: Novartis Pharmaceuticals Corporation.

[B9] BLINCYTO^®^ (2014). *Prescribing Information Approved by the U.S. Food and Drug Administration.* (U.S. License No. 1080). Thousand Oaks, CA: Amgen Inc.

[B10] Borgo B. Havranek J. J. (2012). Automated selection of stabilizing mutations in designed and natural proteins. *Proc. Natl. Acad. Sci. U.S.A.* 109 1494–1499. 10.1073/pnas.1115172109 22307603 PMC3277135

[B11] Brennan D. J. O’Connor D. P. Rexhepaj E. Ponten F. Gallagher W. M. (2010). Antibody-based proteomics: fast-tracking molecular diagnostics in oncology. *Nat. Rev. Cancer* 10 605–617. 10.1038/nrc2902 20720569

[B12] Brinkmann U. Reiter Y. Jung S. H. Lee B. Pastan I. (1993). A recombinant immunotoxin containing a disulfide-stabilized Fv fragment. *Proc. Natl. Acad. Sci. U.S.A.* 90 7538–7542. 10.1073/pnas.90.16.7538 8356052 PMC47177

[B13] Buck P. M. Kumar S. Wang X. Agrawal N. J. Trout B. L. Singh S. K. (2012). Computational methods to predict therapeutic protein aggregation. *Methods Mol. Biol.* 899 425–451. 10.1007/978-1-61779-921-1_26 22735968

[B14] Bukau B. Horwich A. L. (1998). The Hsp70 and Hsp60 chaperone machines. *Cell* 92 351–366. 10.1016/s0092-8674(00)80928-9 9476895

[B15] CABLIVI^®^ (2019). *Prescribing Information Approved by the U.S. Food and Drug Administration.* (U.S. License No. 2085). Ghent: Ablynx NV.

[B16] Cai Y. Zhang J. Lao X. Jiang H. Yu Y. Deng Y. (2016). Construction of a disulfide-stabilized diabody against fibroblast growth factor-2 and the inhibition activity in targeting breast cancer. *Cancer Sci.* 107 1141–1150. 10.1111/cas.12981 27251178 PMC4982589

[B17] Chao G. Lau W. L. Hackel B. J. Sazinsky S. L. Lippow S. M. Wittrup K. D. (2006). Isolating and engineering human antibodies using yeast surface display. *Nat. Protoc.* 1 755–768. 10.1038/nprot.2006.94 17406305

[B18] Chartrain M. Chu L. (2008). Development and production of commercial therapeutic monoclonal antibodies in Mammalian cell expression systems: an overview of the current upstream technologies. *Curr. Pharm. Biotechnol.* 9 447–467. 10.2174/138920108786786367 19075685

[B19] Chevalier A. Silva D. A. Rocklin G. J. Hicks D. R. Vergara R. Murapa P. (2017). Massively parallel de novo protein design for targeted therapeutics. *Nature* 550 74–79. 10.1038/nature23912 28953867 PMC5802399

[B20] Choi S. I. Han K. S. Kim C. W. Ryu K. S. Kim B. H. Kim K. H. (2008). Protein solubility and folding enhancement by interaction with RNA. *PLoS One* 3:e2677. 10.1371/journal.pone.0002677 18628952 PMC2444022

[B21] CIMZIA^®^ (2008). *Prescribing Information Approved by the U.S. Food and Drug Administration.* (US License No. 1736). Smyrna, GE: UCB, Inc.

[B22] Conroy P. J. Hearty S. Leonard P. O’Kennedy R. J. (2009). Antibody production, design and use for biosensor-based applications. *Semin. Cell Dev. Biol.* 20 10–26. 10.1016/j.semcdb.2009.01.010 19429487

[B23] de la Cruz Edmonds M. C. Tellers M. Chan C. Salmon P. Robinson D. K. Markusen J. (2006). Development of transfection and high-producer screening protocols for the CHOK1SV cell system. *Mol. Biotechnol.* 34 179–190. 10.1385/mb:34:2:179 17172663

[B24] Demarest S. J. Chen G. Kimmel B. E. Gustafson D. Wu J. Salbato J. (2006). Engineering stability into *Escherichia coli* secreted Fabs leads to increased functional expression. *Protein Eng. Design Select.* 19 325–336. 10.1093/protein/gzl016 16672248

[B25] Demarest S. J. Glaser S. M. (2008). Antibody therapeutics, antibody engineering, and the merits of protein stability. *Curr. Opin. Drug Discov. Dev.* 11 675–687. 18729019

[B26] DePalma A. (2014). Advances in protein expression. *Genet. Eng. Biotechnol. News* 34 24–25. 10.1089/gen.34.01.14

[B27] Dudgeon K. Rouet R. Kokmeijer I. Schofield P. Stolp J. Langley D. (2012). General strategy for the generation of human antibody variable domains with increased aggregation resistance. *Proc. Natl. Acad. Sci. U.S.A.* 109 10879–10884. 10.1073/pnas.1202866109 22745168 PMC3390889

[B28] Ewert S. Honegger A. Pluckthun A. (2003). Structure-based improvement of the biophysical properties of immunoglobulin VH domains with a generalizable approach. *Biochemistry* 42 1517–1528. 10.1021/bi026448p 12578364

[B29] Ewert S. Honegger A. Pluckthun A. (2004). Stability improvement of antibodies for extracellular and intracellular applications: CDR grafting to stable frameworks and structure-based framework engineering. *Methods* 34 184–199. 10.1016/j.ymeth.2004.04.007 15312672

[B30] Famm K. Hansen L. Christ D. Winter G. (2008). Thermodynamically stable aggregation-resistant antibody domains through directed evolution. *J. Mol. Biol.* 376 926–931. 10.1016/j.jmb.2007.10.075 18199455

[B31] Feige M. J. Groscurth S. Marcinowski M. Shimizu Y. Kessler H. Hendershot L. M. (2009). An unfolded CH1 domain controls the assembly and secretion of IgG antibodies. *Mol. Cell.* 34 569–579. 10.1016/j.molcel.2009.04.028 19524537 PMC2908990

[B32] Frenzel A. Hust M. Schirrmann T. (2013). Expression of recombinant antibodies. *Front. Immunol.* 4:217. 10.3389/fimmu.2013.00217 23908655 PMC3725456

[B33] Gaciarz A. Ruddock L. W. (2017). Complementarity determining regions and frameworks contribute to the disulfide bond independent folding of intrinsically stable scFv. *PLoS One* 12:e0189964. 10.1371/journal.pone.0189964 29253024 PMC5734687

[B34] Goldenzweig A. Fleishman S. J. (2018). Principles of protein stability and their application in computational design. *Annu. Rev. Biochem.* 87 105–129. 10.1146/annurev-biochem-062917-12102 29401000

[B35] Graff C. P. Chester K. Begent R. Wittrup K. D. (2004). Directed evolution of an anti-carcinoembryonic antigen scFv with a 4-day monovalent dissociation half-time at 37 degrees C. *Protein Eng. Design Select.* 17 293–304. 10.1093/protein/gzh038 15115853

[B36] Grilo A. L. Mantalaris A. (2019). The increasingly human and profitable monoclonal antibody market. *Trends Biotechnol.* 37 9–16. 10.1016/j.tibtech.2018.05.014 29945725

[B37] Gupta S. K. Shukla P. (2017). Microbial platform technology for recombinant antibody fragment production: a review. *Crit. Rev. Microbiol.* 43 31–42. 10.3109/1040841X.2016.1150959 27387055

[B38] Hartl F. U. Hayer-Hartl M. (2002). Molecular chaperones in the cytosol: from nascent chain to folded protein. *Science* 295 1852–1858. 10.1126/science.1068408 11884745

[B39] Hendrick J. P. Hartl F. U. (1995). The role of molecular chaperones in protein folding. *FASEB J.* 9 1559–1569. 10.1096/fasebj.9.15.8529835 8529835

[B40] Holliger P. Hudson P. J. (2005). Engineered antibody fragments and the rise of single domains. *Nat. Biotechnol.* 23 1126–1136. 10.1038/nbt1142 16151406

[B41] Holliger P. Prospero T. Winter G. (1993). “Diabodies”: small bivalent and bispecific antibody fragments. *Proc. Natl. Acad. Sci. U.S.A.* 90 6444–6448. 10.1073/pnas.90.14.6444 8341653 PMC46948

[B42] Holms W. H. (1986). The central metabolic pathways of *Escherichia coli*: relationship between flux and control at a branch point, efficiency of conversion to biomass, and excretion of acetate. *Curr. Top. Cell Regul.* 28 69–105. 10.1016/b978-0-12-152828-7.50004-4 3098503

[B43] Hsu H. J. Lee K. H. Jian J. W. Chang H. J. Yu C. M. Lee Y. C. (2014). Antibody variable domain interface and framework sequence requirements for stability and function by high-throughput experiments. *Structure* 22 22–34. 10.1016/j.str.2013.10.006 24268647

[B44] Hu W. Xiang J. Y. Kong P. Liu L. Xie Q. Xiang H. (2017). Expression and characterization of a single-chain variable fragment against human LOX-1 in *Escherichia coli* and *Brevibacillus choshinensis*. *J. Microbiol. Biotechnol.* 27 965–974. 10.4014/jmb.1702.02007 28274103

[B45] Huang Y. Li Y. Wang Y. G. Gu X. Wang Y. Shen B. F. (2007). An efficient and targeted gene integration system for high-level antibody expression. *J. Immunol. Methods* 322 28–39. 10.1016/j.jim.2007.01.022 17350648

[B46] Humphreys D. P. (2003). Production of antibodies and antibody fragments in *Escherichia coli* and a comparison of their functions, uses and modification. *Curr. Opin. Drug Discov. Dev.* 6 188–196. 12669454

[B47] Ionescu R. M. Vlasak J. Price C. Kirchmeier M. (2008). Contribution of variable domains to the stability of humanized IgG1 monoclonal antibodies. *J. Pharm. Sci.* 97 1414–1426. 10.1002/jps.21104 17721938

[B48] Jager M. Pluckthun A. (1999a). Domain interactions in antibody Fv and scFv fragments: effects on unfolding kinetics and equilibria. *FEBS Lett.* 462 307–312. 10.1016/s0014-5793(99)01532-x 10622716

[B49] Jager M. Pluckthun A. (1999b). Folding and assembly of an antibody Fv fragment, a heterodimer stabilized by antigen. *J. Mol. Biol.* 285 2005–2019. 10.1006/jmbi.1998.2425 9925781

[B50] Jefferis R. (2009). Glycosylation as a strategy to improve antibody-based therapeutics. *Nat. Rev. Drug Discov.* 8 226–234. 10.1038/nrd2804 19247305

[B51] Jespers L. Schon O. Famm K. Winter G. (2004). Aggregation-resistant domain antibodies selected on phage by heat denaturation. *Nat. Biotechnol.* 22 1161–1165. 10.1038/nbt1000 15300256

[B52] Jordan J. L. Arndt J. W. Hanf K. Li G. Hall J. Demarest S. (2009). Structural understanding of stabilization patterns in engineered bispecific Ig-like antibody molecules. *Proteins* 77 832–841. 10.1002/prot.22502 19626705

[B53] Kang T. H. Jung S. T. (2019). Boosting therapeutic potency of antibodies by taming Fc domain functions. *Exp. Mol. Med.* 51 1–9. 10.1038/s12276-019-0345-9 31735912 PMC6859160

[B54] Kang T. H. Jung S. T. (2020). Reprogramming the constant region of immunoglobulin G subclasses for enhanced therapeutic potency against cancer. *Biomolecules* 10:382. 10.3390/biom10030382 32121592 PMC7175108

[B55] Khodabakhsh F. Zia M. F. Moazen F. Rabbani M. Sadeghi H. M. (2013). Comparison of the cytoplasmic and periplasmic production of reteplase in *Escherichia coli*. *Prep. Biochem. Biotechnol.* 43 613–623. 10.1080/10826068.2013.764896 23768109

[B56] Kim Y. S. Son A. Kim J. Kwon S. B. Kim M. H. Kim P. (2018). Chaperna-mediated assembly of ferritin-based middle east respiratory syndrome-coronavirus nanoparticles. *Front. Immunol.* 9:1093. 10.3389/fimmu.2018.01093 29868035 PMC5966535

[B57] Kreitman R. J. Pastan I. (2011). Antibody fusion proteins: anti-CD22 recombinant immunotoxin moxetumomab pasudotox. *Clin. Cancer Res.* 17 6398–6405. 10.1158/1078-0432.CCR-11-0487 22003067 PMC3201735

[B58] Kunert R. Reinhart D. (2016). Advances in recombinant antibody manufacturing. *Appl. Microbiol. Biotechnol.* 100 3451–3461. 10.1007/s00253-016-7388-9 26936774 PMC4803805

[B59] Kuroda D. Shirai H. Jacobson M. P. Nakamura H. (2012). Computer-aided antibody design. *Protein Eng. Design Select.* 25 507–521. 10.1093/protein/gzs024 22661385 PMC3449398

[B60] Lawrence M. S. Phillips K. J. Liu D. R. (2007). Supercharging proteins can impart unusual resilience. *J. Am. Chem. Soc.* 129 10110–10112. 10.1021/ja071641y 17665911 PMC2820565

[B61] Le Gall F. Reusch U. Little M. Kipriyanov S. M. (2004). Effect of linker sequences between the antibody variable domains on the formation, stability and biological activity of a bispecific tandem diabody. *Protein Eng. Des. Sel.* 17 357–366. 10.1093/protein/gzh039 15126676

[B62] Lee J. Der B. S. Karamitros C. S. Li W. Marshall N. M. Lungu O. I. (2019). Computer-based engineering of thermostabilized antibody fragments. *AIChE J.* 66:e16864. 10.1002/aic.16864 32336757 PMC7181397

[B63] Levy R. Ahluwalia K. Bohmann D. J. Giang H. M. Schwimmer L. J. Issafras H. (2013). Enhancement of antibody fragment secretion into the *Escherichia coli* periplasm by co-expression with the peptidyl prolyl isomerase, FkpA, in the cytoplasm. *J. Immunol. Methods* 394 10–21. 10.1016/j.jim.2013.04.010 23624043

[B64] Liu C. Dalby B. Chen W. Kilzer J. M. Chiou H. C. (2008). Transient transfection factors for high-level recombinant protein production in suspension cultured mammalian cells. *Mol. Biotechnol.* 39 141–153. 10.1007/s12033-008-9051-x 18327552

[B65] Liu Z. Zhang J. Fan H. Yin R. Zheng Z. Xu Q. (2015). Expression and purification of soluble single-chain Fv against human fibroblast growth factor receptor 3 fused with Sumo tag in *Escherichia coli*. *Electron. J. Biotechnolo.* 18 302–306. 10.1016/j.ejbt.2015.05.006

[B66] Lobstein J. Emrich C. A. Jeans C. Faulkner M. Riggs P. Berkmen M. (2012). SHuffle, a novel *Escherichia coli* protein expression strain capable of correctly folding disulfide bonded proteins in its cytoplasm. *Microb Cell Fact* 11:56. 10.1186/1475-2859-11-56 22569138 PMC3526497

[B67] LUCENTIS^®^ (2006). *Prescribing Information Approved by the U.S. Food and Drug Administration.* South San Francisco, CA: Genentech Inc.

[B68] LUMOXITI^TM^ (2018). *PRESCRIBING INFORMATION Approved by the U.S. Food and Drug Administration (U.S. License No. 2059).* Wilmington: AstraZeneca Pharmaceuticals LP.

[B69] Mack M. Riethmuller G. Kufer P. (1995). A small bispecific antibody construct expressed as a functional single-chain molecule with high tumor cell cytotoxicity. *Proc. Natl. Acad. Sci. U.S.A.* 92 7021–7025. 10.1073/pnas.92.15.7021 7624362 PMC41463

[B70] Makino T. Skretas G. Kang T. H. Georgiou G. (2011). Comprehensive engineering of *Escherichia coli* for enhanced expression of IgG antibodies. *Metab. Eng.* 13 241–251. 10.1016/j.ymben.2010.11.002 21130896 PMC3057344

[B71] Marcatili P. Rosi A. Tramontano A. (2008). PIGS: automatic prediction of antibody structures. *Bioinformatics* 24 1953–1954. 10.1093/bioinformatics/btn341 18641403

[B72] McConnell A. D. Spasojevich V. Macomber J. L. Krapf I. P. Chen A. Sheffer J. C. (2013). An integrated approach to extreme thermostabilization and affinity maturation of an antibody. *Protein Eng. Design Select.* 26 151–164. 10.1093/protein/gzs090 23173178

[B73] McConnell A. D. Zhang X. Macomber J. L. Chau B. Sheffer J. C. Rahmanian S. (2014). A general approach to antibody thermostabilization. *mAbs* 6 1274–1282. 10.4161/mabs.29680 25517312 PMC4623350

[B74] McKenna R. Lombana T. N. Yamada M. Mukhyala K. Veeravalli K. (2019). Engineered sigma factors increase full-length antibody expression in *Escherichia coli*. *Metab. Eng.* 52 315–323. 10.1016/j.ymben.2018.12.009 30610917

[B75] Miklos A. E. Kluwe C. Der B. S. Pai S. Sircar A. Hughes R. A. (2012). Structure-based design of supercharged, highly thermoresistant antibodies. *Chem. Biol.* 19 449–455. 10.1016/j.chembiol.2012.01.018 22520751 PMC5583727

[B76] Miller B. R. Demarest S. J. Lugovskoy A. Huang F. Wu X. Snyder W. B. (2010). Stability engineering of scFvs for the development of bispecific and multivalent antibodies. *Protein Eng. Design Select.* 23 549–557. 10.1093/protein/gzq028 20457695

[B77] Mizukami M. Onishi H. Hanagata H. Miyauchi A. Ito Y. Tokunaga H. (2018). Efficient production of Trastuzumab Fab antibody fragments in Brevibacillus choshinensis expression system. *Protein Expr. Purif.* 150 109–118. 10.1016/j.pep.2018.05.013 29857036

[B78] Montoliu-Gaya L. Martinez J. C. Villegas S. (2017). Understanding the contribution of disulfide bridges to the folding and misfolding of an anti-Abeta scFv. *Protein Sci.* 26 1138–1149. 10.1002/pro.3164 28340507 PMC5441429

[B79] Nieba L. Honegger A. Krebber C. Pluckthun A. (1997). Disrupting the hydrophobic patches at the antibody variable/constant domain interface: improved in vivo folding and physical characterization of an engineered scFv fragment. *Protein Eng.* 10 435–444. 10.1093/protein/10.4.435 9194169

[B80] Petrus M. L. C. Kiefer L. A. Puri P. Heemskerk E. Seaman M. S. Barouch D. H. (2019). A microbial expression system for high-level production of scFv HIV-neutralizing antibody fragments in *Escherichia coli*. *Appl. Microbiol. Biotechnol.* 103 8875–8888. 10.1007/s00253-019-10145-10141 31641814 PMC6851033

[B81] Power B. E. Hudson P. J. (2000). Synthesis of high avidity antibody fragments (scFv multimers) for cancer imaging. *J. Immunol. Methods* 242 193–204. 10.1016/s0022-1759(00)00201-5 10986400

[B82] PRAXBIND^®^ (2015). *Prescribing Information Approved by the U.S. Food and Drug Administration.* (US License No. 2006). Ingelheim am Rhein: Boehringer Ingelheim Pharmaceuticals Inc.

[B83] ReoPro^®^ (1994). *Prescribing Information Approved by the U.S. Food and Drug Administration.* (U.S. License Number: 1864). Horsham: Janssen Biotech, Inc.

[B84] Richards D. A. Maruani A. Chudasama V. (2017). Antibody fragments as nanoparticle targeting ligands: a step in the right direction. *Chem. Sci.* 8 63–77. 10.1039/c6sc02403c 28451149 PMC5304706

[B85] Robinson M. P. Ke N. Lobstein J. Peterson C. Szkodny A. Mansell T. J. (2015). Efficient expression of full-length antibodies in the cytoplasm of engineered bacteria. *Nat. Commun.* 6:8072. 10.1038/ncomms9072 26311203 PMC4560801

[B86] Rosano G. L. Ceccarelli E. A. (2014). Recombinant protein expression in *Escherichia coli*: advances and challenges. *Front. Microbiol.* 5:172. 10.3389/fmicb.2014.00172 24860555 PMC4029002

[B87] Saerens D. Huang L. Bonroy K. Muyldermans S. (2008). Antibody fragments as probe in biosensor development. *Sensors* 8 4669–4686. 10.3390/s8084669 27873779 PMC3705465

[B88] Saunders K. O. (2019). Conceptual approaches to modulating antibody effector functions and circulation half-life. *Front. Immunol.* 10:1296. 10.3389/fimmu.2019.01296 31231397 PMC6568213

[B89] Sevy A. M. Wu N. C. Gilchuk I. M. Parrish E. H. Burger S. Yousif D. (2019). Multistate design of influenza antibodies improves affinity and breadth against seasonal viruses. *Proc. Natl. Acad. Sci. U.S.A.* 116 1597–1602. 10.1073/pnas.1806004116 30642961 PMC6358683

[B90] Sircar A. Kim E. T. Gray J. J. (2009). RosettaAntibody: antibody variable region homology modeling server. *Nucleic Acids Res.* 37 W474–W479. 10.1093/nar/gkp387 19458157 PMC2703951

[B91] Steipe B. (2004). Consensus-based engineering of protein stability: from intrabodies to thermostable enzymes. *Methods Enzymol.* 388 176–186. 10.1016/S0076-6879(04)88016-9 15289071

[B92] Strauch E. M. Bernard S. M. La D. Bohn A. J. Lee P. S. Anderson C. E. (2017). Computational design of trimeric influenza-neutralizing proteins targeting the hemagglutinin receptor binding site. *Nat. Biotechnol.* 35 667–671. 10.1038/nbt.3907 28604661 PMC5512607

[B93] Structural Genomics Consortium, China Structural Genomics Consortium, Northeast Structural Genomics, Consortium, Graslund S. Nordlund P. Weigelt J. (2008). Protein production and purification. *Nat. Methods* 5 135–146. 10.1038/nmeth.f.202 18235434 PMC3178102

[B94] Suresh T. Lee L. X. Joshi J. Barta S. K. (2014). New antibody approaches to lymphoma therapy. *J Hematol. Oncol.* 7:58. 10.1186/s13045-014-0058-4 25355407 PMC4172963

[B95] Teerinen T. Valjakka J. Rouvinen J. Takkinen K. (2006). Structure-based stability engineering of the mouse IgG1 Fab fragment by modifying constant domains. *J. Mol. Biol.* 361 687–697. 10.1016/j.jmb.2006.06.073 16876195

[B96] Terpe K. (2006). Overview of bacterial expression systems for heterologous protein production: from molecular and biochemical fundamentals to commercial systems. *Appl. Microbiol. Biotechnol.* 72 211–222. 10.1007/s00253-006-0465-8 16791589

[B97] Tischenko V. M. Zav’yalov V. P. Medgyesi G. A. Potekhin S. A. Privalov P. L. (1982). A thermodynamic study of cooperative structures in rabbit immunoglobulin G. *Eur. J. Biochem.* 126 517–521. 10.1111/j.1432-1033.1982.tb06811.x 7140745

[B98] Trivedi M. V. Laurence J. S. Siahaan T. J. (2009). The role of thiols and disulfides on protein stability. *Curr. Protein Peptide Sci.* 10 614–625. 10.2174/138920309789630534 19538140 PMC3319691

[B99] Tseng T. T. Tyler B. M. Setubal J. C. (2009). Protein secretion systems in bacterial-host associations, and their description in the gene ontology. *BMC Microbiol.* 9(Suppl. 1):S2. 10.1186/1471-2180-9-S1-S2 19278550 PMC2654662

[B100] Vermeer A. W. Norde W. (2000). The thermal stability of immunoglobulin: unfolding and aggregation of a multi-domain protein. *Biophys. J.* 78 394–404. 10.1016/S0006-3495(00)76602-1 10620303 PMC1300647

[B101] Wang R. Xiang S. Feng Y. Srinivas S. Zhang Y. Lin M. (2013). Engineering production of functional scFv antibody in *E. coli* by co-expressing the molecule chaperone Skp. *Front. Cell Infect. Microbiol.* 3:72. 10.3389/fcimb.2013.00072 24224158 PMC3818579

[B102] Webber K. O. Reiter Y. Brinkmann U. Kreitman R. Pastan I. (1995). Preparation and characterization of a disulfide-stabilized Fv fragment of the anti-Tac antibody: comparison with its single-chain analog. *Mol. Immunol.* 32 249–258. 10.1016/0161-5890(94)00150-y 7723770

[B103] Whitelegg N. R. J. Rees A. R. (2000). WAM: an improved algorithm for modelling antibodies on the WEB. *Protein Eng.* 13 819–824. 10.1093/protein/13.12.819 11239080

[B104] Wong M. S. Wu S. Causey T. B. Bennett G. N. San K. Y. (2008). Reduction of acetate accumulation in *Escherichia coli* cultures for increased recombinant protein production. *Metab. Eng.* 10 97–108. 10.1016/j.ymben.2007.10.003 18164227

[B105] Wu J. Fu J. Zhang M. Liu D. (2015). Blinatumomab: a bispecific T cell engager (BiTE) antibody against CD19/CD3 for refractory acute lymphoid leukemia. *J. Hematol. Oncol.* 8:104. 10.1186/s13045-015-0195-4 26337639 PMC4558758

[B106] Yamauchi S. Kobashigawa Y. Fukuda N. Teramoto M. Toyota Y. Liu C. (2019). Cyclization of Single-Chain Fv antibodies markedly suppressed their characteristic aggregation mediated by inter-chain VH-VL Interactions. *Molecules* 24:2620. 10.3390/molecules24142620 31323851 PMC6681014

[B107] Yang S. W. Jang Y. H. Kwon S. B. Lee Y. J. Chae W. Byun Y. H. (2018). Harnessing an RNA-mediated chaperone for the assembly of influenza hemagglutinin in an immunologically relevant conformation. *FASEB J.* 32 2658–2675. 10.1096/fj.201700747RR 29295864 PMC5901386

[B108] Yusakul G. Nuntawong P. Sakamoto S. Ratnatilaka Na Bhuket P. Kohno T. Kikkawa N. (2017). Bacterial expression of a single-chain variable fragment (scFv) Antibody against ganoderic Acid A: a Cost-effective approach for quantitative analysis using the scFv-Based enzyme-linked immunosorbent assay. *Biol. Pharm. Bull.* 40 1767–1774. 10.1248/bpb.b17-00531 28966249

[B109] Zhang K. Geddie M. L. Kohli N. Kornaga T. Kirpotin D. B. Jiao Y. (2015). Comprehensive optimization of a single-chain variable domain antibody fragment as a targeting ligand for a cytotoxic nanoparticle. *mAbs* 7 42–52. 10.4161/19420862.2014.985933 25484041 PMC4622481

[B110] Zhao J. X. Yang L. Gu Z. N. Chen H. Q. Tian F. W. Chen Y. Q. (2010). Stabilization of the single-chain fragment variable by an interdomain disulfide bond and its effect on antibody affinity. *Intern. J. Mol. Sci.* 12 1–11. 10.3390/ijms12010001 21339972 PMC3039938

